# Gui-zhi decoction for allergic rhinitis: A protocol for systematic review and meta analysis

**DOI:** 10.1097/MD.0000000000021931

**Published:** 2020-09-18

**Authors:** Bin Zou, Wentao Zhang, Yuechuan Cai, Fubing Zhang, Chengrui Wang

**Affiliations:** Otolaryngology department, Leshan hospital of TCM, Sichuan Province, P.R. China.

**Keywords:** allergic rhinitis, clinical trial, gui-zhi decoction, protocol, systematic review

## Abstract

**Background:**

The treatment effects and safety of Gui-zhi decoction (GZD) for patients with allergic rhinitis have yet to be clarified.

**Methods:**

We will search PubMed, EMBASE (Excerpta Medical Database), Cochrane Library, Chinese Cochrane Centre's Controlled Trials Register Platform, Wanfang Chinese Digital Periodical and Conference Database, China National Knowledge Infrastructure Database, and VIP Chinese Science and Technique Journals Database to collect randomized controlled trials of Gui-zhi decoction (GZD) for allergic rhinitis. RevMan5.3 software was used to conduct a meta-analysis. Grading of Recommendations Assessment, Development and Evaluation methodology was applied to evaluate the evidence quality for each outcome. Cochrane Risk Assessment Tool will be used to assess the quality of eligible studies according to the Cochrane handbook.

**Results:**

The results of this systematic review will provide a synthesis of current evidence of GZD and we have a specific opportunity to determine the efficacy and safety of it.

**Conclusion:**

This study will explore the efficacy and safety of GZD to treat allergic rhinitis.

**OSF registration:**

Identifier: DOI 10.17605/OSF.IO/CWQNH (https://osf.io/cwqnh/)

## Abstract 2

Supplemental Digital Content is available in the text

## 1 Introduction

Allergic rhinitis (AR) is defined as chronic inflammation of the nasal mucous membrane, typically induced by immunoglobulin E- (IgE-) mediated sensitization to environmental allergens. These include dust, domestic animals, pollens, and molds. AR is defined by the onset of 2 or more of the following symptoms: nasal discharge, sneezing, nasal itching, and congestion, all of which interfere with activities of daily living, disrupt regular sleep patterns, or exert a negative influence on the patient's social life or intellectual performance. The incidence of AR is approximately 20% to 40% in the US population.^[1]^ A survey of the worldwide pediatric population showed that the morbidity of AR was 10% to 20% in 2015.^[2]^ Subcutaneous injection immunotherapy and sublingual immunotherapy^[3]^ are common treatment modalities for this disease, but the effectiveness of those therapies is in doubt and remains to be demonstrated conclusively.

Guizhi decoction come from Typhoid Theory written by Zhang Zhongjing, a famous physician in the Han dynasty. It is called “the leading group of the group” by later doctors. At present, Guizhi decoction is widely used in treatment of many diseases of internal, external, gynecologic and other diseases.^[4,5]^ Pharmacological experimental studies had shown that Guizhi decoction could play a big role in dual-directional regulation on sweat gland, body temperature, immune function, gastrointestinal peristalsis, and blood pressure, and could also play the role of anti-inflammatory, antibacterial, antiviral, anti-allergic, analgesic, hypoglycemic, and cardiovascular protection.^[6]^

As a classical herbal medicine formula for AR, Gui-zhi decoction (GZD) and other herbal medicine formulas as effective interventions have been recommended in a Clinical Guideline for alleviation of symptoms of AR in 2015.^[7]^ At least 3 reviews of herbal medicine therapy for AR have been located.^[8–10]^ However, these reviews did not consider GZD With the publication of a fair number of trials on GZD for AR in recent years, there is an urgent need for a systematic review to summarize the evidence from all available studies of GZD. Thus, the aim of this review was to evaluate critically the current state of evidence from randomized controlled trials (RCTs) on the use of GZD in patients of AR, according to the guidelines set down in the Cochrane Handbook.

## 2 Methods

This meta-analysis will be based on the preferred reporting items for the systematic review and meta-analysis of the (PRISMA) project.^[11]^

### 2.1 Inclusion criteria for study selection

#### 2.1.1 Type of studies

All the RCTs to explore the specific efficacy and safety of GZD in the treatment of allergic rhinitis will be included. Cross-trials, quasi-RCT, case reports, observation study, animal study, repeatedly published studies, and studies did not have access to complete data will be excluded. If we are unable to find at least 5 eligible RCTs for the systematic review, we will broaden our inclusion criteria to include semi-randomized control studies, non-randomized studies of GZD in allergic rhinitis patients using the Cochrane Effective Practice and Organization of CGZDe (EPOC) approach to categorize the types of studies.^[12]^

#### 2.1.2 Types of participants

Participants who meet the diagnostic criteria of allergic rhinitis were all included. However, allergic rhinitis merged with other food allergy or allergic asthma or allergic conjunctivitis and other allergic diseases were excluded. This was done because targeted drug combination methods in these studies could not be used to compare the effects. All included participants in this review regardless of their age, race, and gender.

#### 2.1.3 Types of outcome measures

Trials will be required to include as outcome measures either relief of symptoms of AR or evaluation of the efficacy of GZD in AR. Other important clinical outcomes included recurrence rate, influence on quality of life, improvement in symptom scoring, and adverse events.

The efficacy of GZD for AR, improvement in quality of life, and improvement of symptom scoring were set as primary outcomes. Recurrence rate and adverse events (such as dry mouth, headache, hypersomnia, palpitations, and gastrointestinal discomfort) were set as secondary outcomes.

### 2.2 Search methods for identification of studies

#### 2.2.1 Data sources

A total of 6 databases were searched: PubMed (1992 to May 8, 2021), EMBASE (Excerpta Medical Database) (1992 to May 8, 2021), Cochrane Library (Issue 9 of May 8, 2021), Chinese Cochrane Centre's Controlled Trials Register Platform (up to May 8, 2021), Wanfang ChineseDigital Periodical and Conference Database (1997 to May 8, 2021), China National Knowledge Infrastructure (CNKI) Database (1992 to May 8, 2021), and VIP Chinese Science and Technique Journals Database (1992 to May 8, 2021). Besides, Chinese Clinical Trial Registry Center was also retrieved for ongoing trials.

#### 2.2.2 Searching other resources

Chinese Clinical Trial Registry Center will also be screened for ongoing trials. We will also review the references of included manuscripts to identify any information about missed trials. We will contact the author if we cannot identify information from the data.

#### 2.2.3 Search strategy

We will employ a broad electronic search strategy in Supplemental Digital Content (Appendix A, http://links.lww.com/MD/E821).

### 2.3 Data extraction, quality and validation

#### 2.3.1 Study selection and inclusion

**Figure 1:**
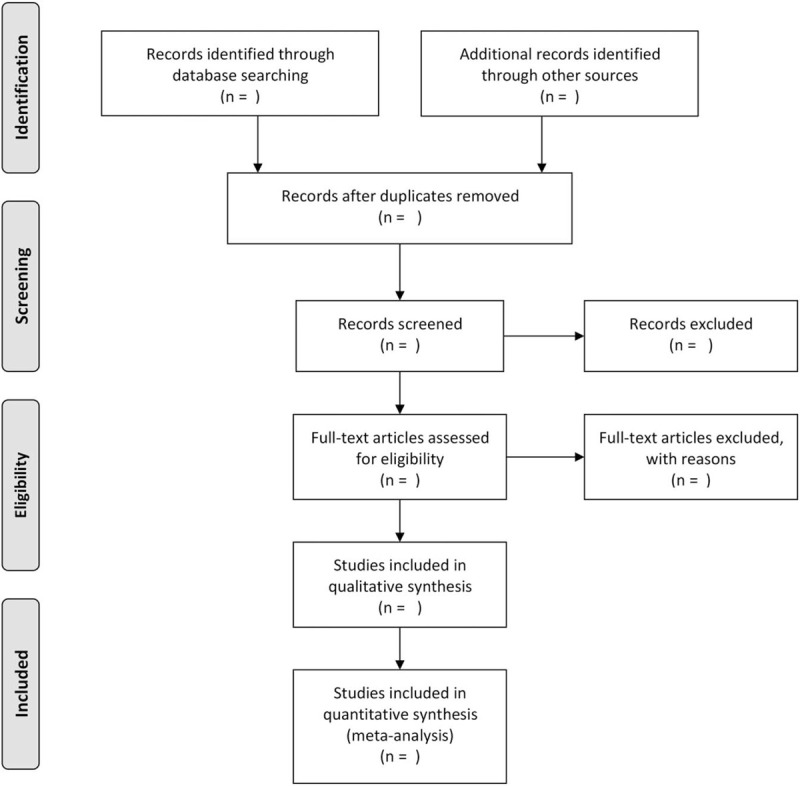
Flow diagram of study selection process.

Researchers will import the literature retrieved to the Endnote X7 and eliminate the duplicate data. All titles and abstracts returned using the search strategy above will be screened by 2 independent investigators (WTZ, FBZ) in line with our advanced inclusion criteria. And then, the full text of the entire study will be reviewed by 3 authors for analysis. Any differences will be resolved by consensus. Finally, another study member will resolve the inconsistencies and check the final literature that will be included. Study selection process will be shown on Figure 1.

#### 2.3.2 Data extraction and management

The raw data from the papers will be extracted separately by 3 authors and will include: author details, publication information, sample size, and original study design information, such as intervention and comparison (dose, route, and time), outcome measures, and follow-up information. All extracted data will be verified by a second investigator to ensure accuracy and completeness. All outcome variables will be collected, regardless of the number of studies that the outcome assessed. If conflict, arbitration will be conducted through discussion or through the third reviewer (FBZ, YCC). Preferred Reporting Items for Systematic Reviews and Meta-Analyses (PRISMA) diagram (Fig. 1) based on the search strategy and eligibility assessment to show the flow of included and excluded studies will be developed by us (BZ, FBZ).

#### 2.3.3 Assessment of risk of bias

The methodological quality of the included RCTs will be assessed based on the instrument developed in the Cochrane Handbook for Systematic of Interventions by 3 investigators. The tool evaluates studies based on 7 criteria:

(1) randomization generation,
(2) allocation concealment,
(3) blinding of outcome assessors,
(4) blinding patients/study personnel,
(5) incomplete outcome data (that is, lost to follow-up),
(6) selective outcome reporting, and
(7) other risks of bias.

We will define other bias as trials which may be sponsored by GZD manufacturers, and in which baseline characteristics are not similar between the different intervention groups. We will also assess publication bias by examining funnel plots if there are 10 or more trials reporting the primary outcomes.

### 2.4 Quantitative data and statistical methods

#### 2.4.1 Quantitative data synthesis

We will analyzed outcomes by intention-to-treat (ITT). In cases of multiple reports of the same trial, we will use all relevant data and analyse it as a single study. We pooled summary measures using DerSimonian and Laird random-effects, estimating heterogeneity using the Mantel-Haenszel Model. For dichotomous outcomes, we combined data using risk ratio and if the outcome could happen more than once in the same continuous outcomes across studies using the mean difference, or the standardised mean difference if the outcomes were measured with different scales.

#### 2.4.2 Assessment of heterogeneity

In our review, *X*^2^ (threshold *P* = .10) and quantified it using *I*^2^ will be used to assess inter-study heterogeneity.

#### 2.4.3 Assessment of reporting bias

We will attempt to assess publication bias by inspecting funnel plots, statistically by the Harbord modification of Egger test.

### 2.5 Subgroup analysis and investigation of heterogeneity

Prespecified subgroup analyses for the main outcomes included analysis by median age, confirmation of allergic rhinitis at study entry by food challenge, duration of GZD, strating and target dose, and versus controlled drug or placebo or no GZD. We also evaluated outcomes according to which of the 2 phases-build or maintenance-allergic reactions occurred. Post-hoc analyses were by assignment of the control groups to either placebo or avoidence, and by entry and exit challenge threahold.

### 2.6 Sensitivity analysis

Sensitivity analyses to test the robustness of the findings included worst-case or various plausible scenarins for missing participants; disregarding excluded participants or missing data (ie, available case analyses); fixed-effect meta-analysis unpublished trials; adjusting potentially overestimated outcomes for trials terminated early by reducing their effect size; restricting anaphylaxis analyses to only those with moderate-to-severe severity; using the more conservative Knapp-Hartung-Sidik-Jonkman random effect mate-analytic method, or potentially more appropriate empirical continuity correction. We used trial sequential analysis to account for multiple testing, and objectively assessed imprecision by examining for sufficient data to avoid type 1 (false-positive) and type 2 (false-negative) errors.

#### 2.6.1 Grading the quality of evidence

We will apply the Grading of Recommendation Assessment, Development and Evaluation method to evaluate the level of confidence in regards to outcomes. Two independent reviewers will conduct the assessment. In most cases, disagreement was resolved by discussion. If disagreement remained after discussion, a third reviewer will be consulted before taking the final decision on the disagreement.

## 3 Discussion

Allergic rhinitis is associated with poor quality of life for both allergic individuals. GZD may have a more advantageous adverse event profile and better adherence to allergic rhinitis. However, clinical benefits and adverse events of GZD for allergic rhinitis is unclear. Therefore, promoting GZD in the clinical treatment of GZD with its acceptability is badly needed.

Moreover, we foresee several potential limitations with this systematic review: heterogeneity of clinical outcomes, substandard quality of existing studies, which are the focus of our project. Therefore, we will present our findings using descriptive methods, if necessary. This study protocol has been designed according to herbal medicine for allergic rhinitis. Our hope is that the dissemination of this protocol will allow us to obtain feedback and constructive criticism of the methods before our study is conducted.

In conclusion, the proposed systematic review will provide insight into the clinical impact of GZD in treatment of allergic rhinitis patients. The results have the potential to inform national and international guidelines on the care and management of GZD in the allergic rhinitis population. The review will also help to highlight areas requiring further rigorously designed research on this topic.

## Author contributions

**Data curation**: Fubing Zhang.

**Formal analysis**: Fubing Zhang.

**Methodology**: Wentao Zhang.

**Project administration**: Chengrui Wang.

**Software**: Chengrui Wang.

**Supervision**: Bin Zou.

**Validation**: Yuechuan Cai.

**Visualization**: Bin Zou.

**Visualization and software**: Bin Zou, Yuechuan Cai.

**Writing – original draft**: Bin Zou.

**Writing – review & editing**: Fubing Zhang.

## Abbreviations

Abbreviations: GZD = Gui-zhi decoction, RCTs = randomized controlled trials.
